# Case Report: The integration of chemoradiotherapy and immunotherapy in a patient with advanced-stage renal squamous cell carcinoma and pulmonary metastases

**DOI:** 10.3389/fimmu.2025.1645909

**Published:** 2025-08-08

**Authors:** Yanlin Niu, Shengchao Wang, Xinzhou Deng, Ming Luo, Jingjing Chai, Zhiguo Luo

**Affiliations:** 1 Department of Oncology, Taihe Hospital, Hubei University of Medicine, Shiyan, Hubei, China; 2 Key Laboratory of Cancer Therapy Resistance and Clinical Translational Study, Shiyan, Hubei, China

**Keywords:** renal squamous cell carcinoma, pulmonary metastases, immunotherapy, radiotherapy, chemotherapy

## Abstract

Renal squamous cell carcinoma (RSCC) is an uncommon malignancy, representing less than 1% of all renal cancers, and is associated with a notably poor prognosis. Surgical intervention is the primary treatment modality for early and intermediate-stage cases; however, the efficacy of combined chemoradiotherapy and immunotherapy in advanced-stage patients remains unclear. In this report, we present a case of RSCC with pulmonary metastases, wherein the patient attained disease remission following a treatment regimen comprising combined chemo-radiotherapy and immunotherapy. The patient demonstrated a progression-free survival (PFS) of 12 months and an overall survival (OS) of 14 months. This case study aims to provide a comprehensive analysis of the treatment approach, potentially serving as a reference for therapeutic strategies in patients with inoperable RSCC.

## 1 Introduction

Renal squamous cell carcinoma (RSCC), a highly uncommon and aggressive variant of renal cell carcinoma (RCC), accounts for less than 1% of all renal malignancies. This subtype is marked by limited therapeutic options and rapid disease progression, leading to a poor prognosis (1). As a subtype of non-clear cell renal cell carcinoma (nccRCC), RSCC demonstrates significant heterogeneity. Notably, nccRCC is distinguished from clear cell renal cell carcinoma (ccRCC) by its unique molecular characteristics and differential treatment responses (2). The development of RSCC is potentially attributable to distinct carcinogenic pathways. Various etiological factors, such as nephrolithiasis, prior radiotherapy, analgesic misuse, and infections, have been implicated in the pathogenesis of squamous cell carcinoma of the upper urinary tract. These factors induce chronic and persistent urothelial injury, leading to squamous metaplasia and dysplasia, which may progress to squamous cell carcinoma (3, 4). Approximately 50% of patients diagnosed with primary renal squamous cell carcinoma (PRSCC) present with concurrent nephrolithiasis or chronic infections. However, the patient in the present case study reported no history of nephrolithiasis, urinary tract infections, or urinary schistosomiasis, indicating the potential involvement of alternative carcinogenic mechanisms. Currently, there is an absence of studies elucidating the specific molecular mechanisms or oncogenes associated with RSCC, leaving its molecular pathogenesis inadequately understood. RSCC is characterized by rapid progression and a propensity for early distant metastasis, which contributes to a poor clinical prognosis. Among patients with renal malignancies, synchronous lung metastasis is the most prevalent pattern of metastasis, followed by metastasis to the bone, lymph nodes, and liver (5). There is presently a paucity of standardized treatment protocols for metastatic nccRCC, with limited large-scale randomized clinical trials available. Consequently, clinical decision-making predominantly relies on small-scale studies and expert consensus (6–8). As one of the rarest subtypes of nccRCC, RSCC suffers from a particularly limited body of evidence-based medical research, further complicating its treatment (9). In this report, we present the first documented case of RSCC complicated by pulmonary metastases, which was managed with a combination of chemoradiotherapy and immunotherapy.

## 2 Case report

**Figure 1:**
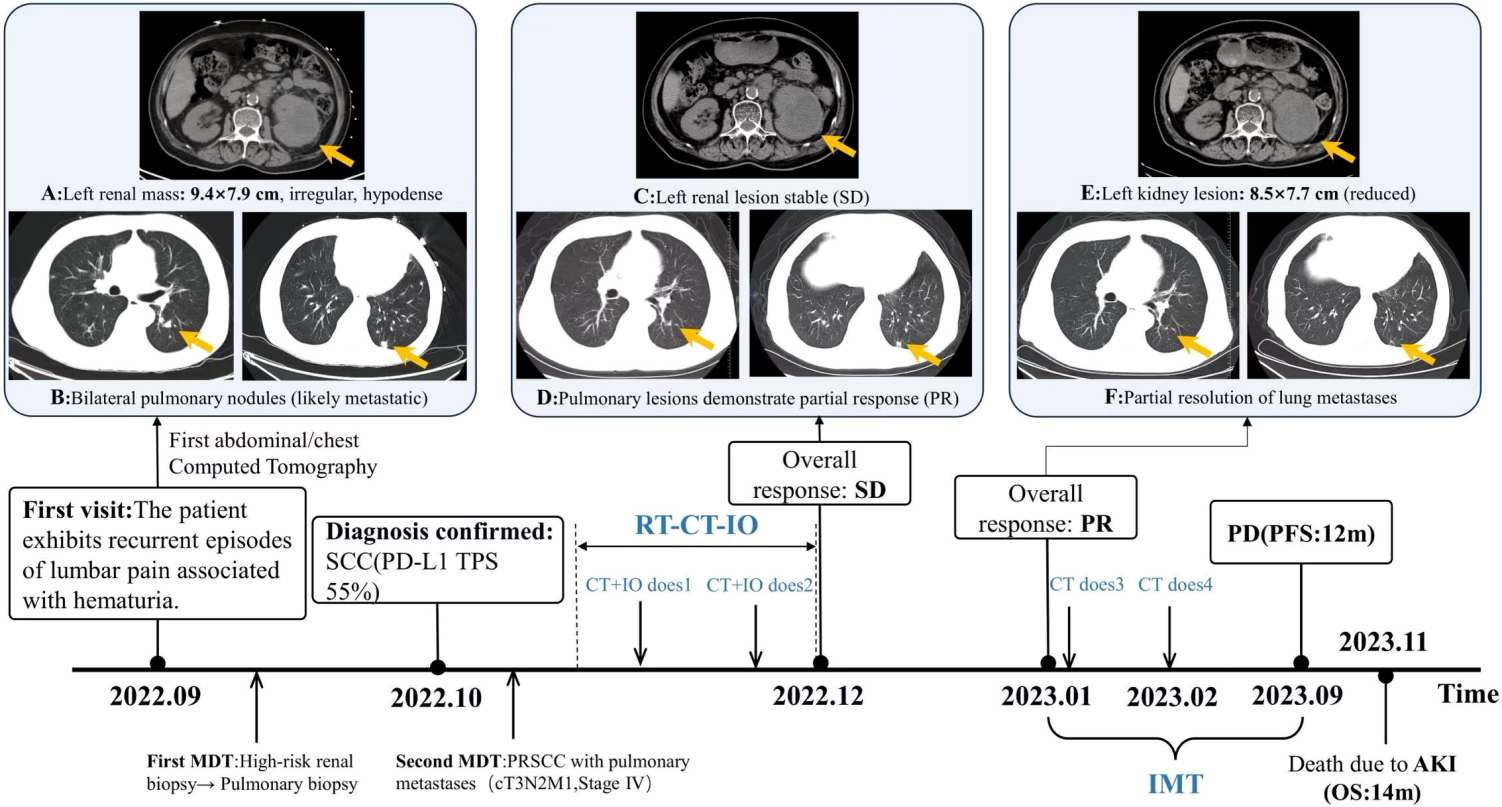
Treatment flowchart and imaging changes during therapy: initial diagnosis (September 2022): **(A)** Contrast-enhanced urological CT: 9.4×7.9 cm irregular low-density mass in left renal parenchyma. **(B)** Chest CT: multiple small bilateral pulmonary nodules. Lung biopsy (October 2022, due to risk) confirmed SCC. Second MDT diagnosed PRSCC with pulmonary metastases. After radiotherapy combined with two cycles of chemo-immunotherapy, December 2022 CT: **(C)** left renal lesion stable; **(D)** bilateral pulmonary nodules significantly decreased. Pre-third chemotherapy, CT re-examination: **(E)** left renal mass reduced to 8.5 × 7.7 cm; **(F)** most pulmonary lesions further regressed, lesion marked by yellow arrow (F left panel) resolved. Patient continued q3w immunotherapy and received two additional nab-paclitaxel cycles. September 2023 re-evaluation: PD. Patient succumbed to AKI (November 2023), OS 14 months. MDT, multidisciplinary team; SCC, squamous cell carcinoma; RSCC, renal squamous cell carcinoma; SD, stable disease; PR, partial response; RT, radiotherapy; CT, chemotherapy; IO, immunotherapy; MT, medical treatment; PFS, progression-free survival; OS, overall survival; IMT, Immunological Maintenance Therapy; AKI, acute kidney injury. PD, Progressive Disease.

**Figure 2:**
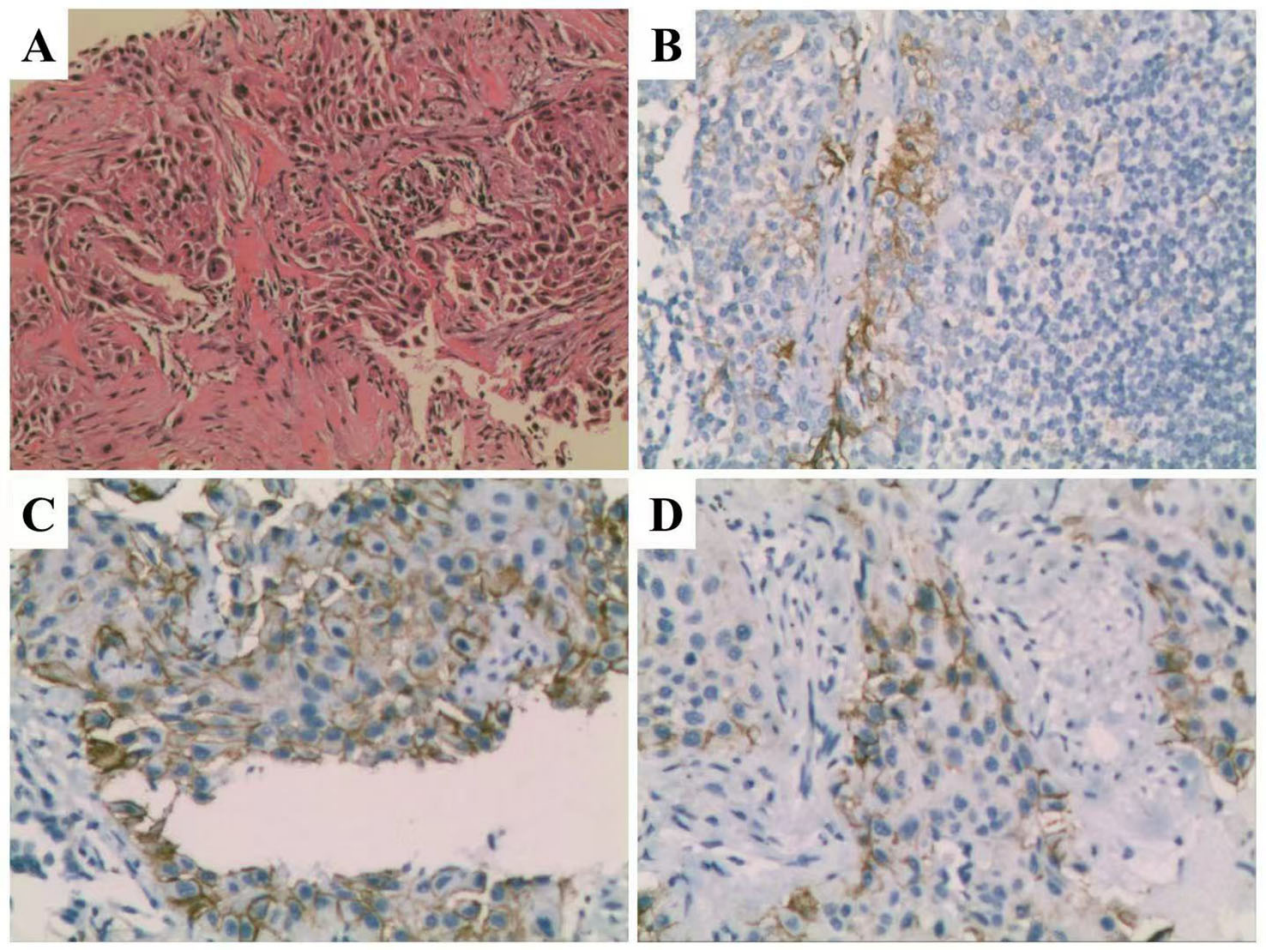
Histopathological examination. **(A)** Hematoxylin and eosin (H&E) staining of a pulmonary metastatic lesion reveals the presence of squamous cell carcinoma. **(B)** Positive control immunohistochemistry (IHC) of pulmonary metastasis using EnVision two-step method. **(C, D)** Assessment of PD-L1 expression in pulmonary metastasis employing the EnVision two-step method.

In September 2022, a 65-year-old male presented with a 2-month history of recurrent flank pain and hematuria. Serum biochemistry was normal, but tumor markers indicated elevated levels: squamous cell carcinoma antigen (SCC) at 2.88 ng/mL (normal 0–1.5 ng/mL), carbohydrate antigen 19-9 (CA 19-9) at 7.13 ng/mL (normal 0–3.4 ng/mL), and cytokeratin 19 fragment (CYFRA 21–1) at 22.9 ng/mL (normal 0–3.3 ng/mL). A contrast-enhanced CT scan showed an irregular hypodense mass (9.4 × 7.9 cm) in the left renal parenchyma, indicating renal carcinoma with intra-abdominal and retroperitoneal lymph node metastases (**Figure 1A**). Thoracic Computed Tomography (CT) identified multiple bilateral pulmonary nodules, suggesting metastases (**Figure 1B**). Color Doppler ultrasound detected left renal vein thrombosis. After the first multidisciplinary team (MDT) discussion, due to the high risk of renal biopsy, a CT-guided percutaneous lung biopsy was conducted in October 2022. Histopathological analysis confirmed the presence of squamous cell carcinoma (**Figure 2A**), with a programmed death-ligand 1 (PD-L1) tumor proportion score (TPS) of 55% (**Figures 2B–D**). Immunohistochemical staining revealed the following profile: TTF-1(SPT24)(-),NapsinA(-), P40(+),ALK(-),CK(P)(+),Vimentin(+),CK7(+),WT-1(-),PAX8(-). Following a second MDT discussion, a comparative analysis of metastatic patterns and clinical manifestations strongly indicated a primary renal malignancy originating in the left kidney, with pulmonary metastases. Additionally, concurrent intra-abdominal and retroperitoneal lymph node metastases were confirmed. A comprehensive evaluation classified the disease as advanced stage, specifically cT3N2M1, stage IV, according to the 8th edition of the American Joint Committee on Cancer (AJCC) TNM staging system for renal carcinoma.

**Figure 3:**
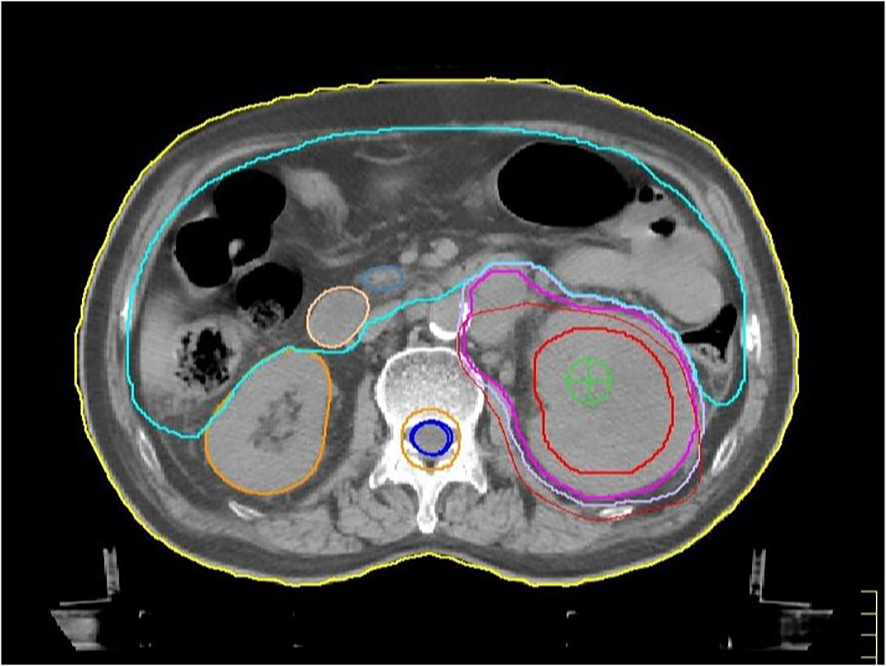
Delineation of radiation target volume. The axial enhanced CT image at the level of the largest diameter of the renal primary lesion, as planned using the Pinnacle system, shows the following: The Gross Tumor Volume (GTV) is outlined by the red solid line, indicating a 9.4×7.9 cm primary lesion in the left kidney. The Clinical Target Volume (CTV) is delineated by the purple solid line, representing the GTV expanded by 10 mm to include the high-risk retroperitoneal lymph node area. The prescribed dose is 60 Gy to the CTV, delivered in 30 fractions.

The patient is a practitioner of Chinese medicine with a decade-long history of hypertension, during which his systolic blood pressure reached a peak of 220 mmHg. His hypertension is effectively managed with amlodipine and metoprolol. He also has an 8-year history of type 2 diabetes, currently treated with metformin, although the status of his glycemic control is not documented. Additionally, the patient has a history of coronary artery disease and takes aspirin on an intermittent basis. He has a smoking history spanning over 20 years, during which he smoked 40 cigarettes daily, but he ceased smoking 10 years ago. Furthermore, he has a history of alcohol consumption exceeding 30 years at a rate of 100 ml per day, but he discontinued alcohol use six months ago. The patient denies any history of infectious diseases, including hepatitis and tuberculosis, as well as any history of surgical procedures, trauma, or blood transfusions. He reports no known food or drug allergies and has no family history of cancer. In light of the limited incidence of advanced RSCC cases and the lack of extensive randomized controlled trials, in conjunction with the patient’s comorbid conditions—namely hypertension, diabetes mellitus, and coronary artery disease—which contraindicate surgical intervention, the second MDT consensus meeting devised a treatment plan consistent with the biological characteristics of squamous carcinoma. This plan involved the integration of chemoradiotherapy and immunotherapy. Financial limitations restricted the patient’s access to category I recommended agents as per the Chinese Society of Clinical Oncology (CSCO)/National Comprehensive Cancer Network (NCCN) guidelines (pembrolizumab/nivolumab), leading to the selection of tislelizumab as the immunotherapeutic agent. Concurrently, to manage ongoing flank pain and gross hematuria, the patient received localized radiotherapy (60 Gy over 30 fractions) directed at the primary renal lesion (**Figure 3**) from October to December 2022. During the course of radiotherapy, the patient was administered two cycles of combination therapy, consisting of nanoparticle albumin-bound paclitaxel (0.3 g intravenously on day 1 every 3 weeks) and tislelizumab (200 mg intravenously on day 1 every 3 weeks). In accordance with the NCCN guidelines, prophylactic administration of granulocyte colony-stimulating factor was implemented for the patient, as the albumin-bound paclitaxel utilized is part of a high-risk chemotherapy regimen. Consequently, no myelosuppression was observed throughout the treatment period. The patient experienced only mild nausea and vomiting, classified as Grade 1, which improved following antiemetic treatment with tropisetron. No adverse reactions related to radiotherapy were noted during this time. Following this therapeutic regimen, a chest CT scan conducted at discharge in December 2022 demonstrated a significant regression of bilateral pulmonary lesions compared to baseline imaging from September 2022 (**Figure 1D**). Concurrently, an abdominal CT scan revealed stability in the primary renal lesions (**Figure 1C**). According to RECIST 1.1 criteria, the pulmonary response was classified as a partial response (PR), with systemic disease stabilization (SD).

In January 2023, a follow-up thoracic CT scan showed further regression of the lesions (**Figure 1F**), including the complete resolution of select metastases (left panel, **Figure 1F**). Abdominal imaging indicated a reduction in the size of the left renal mass to 8.5 × 7.7 cm, along with the resolution of hematuria and flank pain. Consequently, the overall response was upgraded to a PR, leading to the continuation of the original regimen: two additional cycles of nanoparticle albumin-bound paclitaxel (0.3 g IV on day 1, every three weeks) in combination with tislelizumab (200 mg IV on day 1, every three weeks). Subsequent maintenance immunotherapy with tislelizumab (200 mg every three weeks) was administered at a local hospital. During the immunotherapy period, there were no observed adverse reactions, including immune nephritis, immune pneumonia, immune hepatitis, hematuria, endocrine disorders, or dermatological issues.

By September 2023, the patient experienced recurrent gross hematuria subsequent to the self-administration of traditional Chinese medicine. Laboratory analyses revealed acute kidney injury, characterized by a serum creatinine level of 567.1 μmol/L (reference range: 44-120 μmol/L), a potassium level of 5.91 mmol/L (reference range: 3.5-5.3 mmol/L), and an elevated SCC of 8 ng/mL. The most recent computed tomography scan conducted at our institution demonstrated sustained pulmonary improvement; however, it also indicated a progressive enlargement of over 20% in the left renal hilar lymph nodes and ipsilateral adrenal nodules, exhibiting heterogeneous enhancement, which met the Response Evaluation Criteria In Solid Tumors version 1.1 (RECIST v1.1) criteria for Progressive Disease (PD). The patient achieved a progression-free survival (PFS) of 12 months. Within 48 hours, the patient deteriorated to anuria accompanied by worsening azotemia (estimated glomerular filtration rate [eGFR] of 8.25 mL/min/1.73 m²; reference range: 80-120), necessitating intensive care unit management and nephrology intervention. The clinical trajectory ultimately resulted in end-stage renal disease (ESRD), requiring dialysis. Two months later, the patient succumbed to multi-organ failure secondary to acute renal failure, resulting in an overall survival (OS) of 14 months. (For a comprehensive overview of the entire treatment course, please consult **Table 1**).

**Table 1:** Timeline of treatment and clinical outcomes.

Time	Clinical Symptoms/Signs	Treatment Regimen	Adverse Reactions	Efficacy Evaluation
2022-09	Left lumbago, gross hematuria/mild left renal region percussion pain	Diagnosis: CT-guided lung biopsy	Mild pain at biopsy site (Grade 1)	Baseline imaging: Left renal mass 9.4×7.9 cm Multiple pulmonary metastases
2022-10 — 2022-12	Persistent lumbago, alleviated hematuria	Radiotherapy: Left renal primary lesion 60 Gy/30 fractions Chemotherapy: Nab-paclitaxel 0.3g d1 q3w ×2 cycles Immunotherapy: Tislelizumab 200mg d1 q3w ×2 cycles	Grade 1 nausea (relieved by tropisetron) No myelosuppression (due to prophylactic colony-stimulating factor administration)	2022–12 CT: Pulmonary metastases shrank → PR Renal primary lesion stable → SD
2023-01	Lumbago relieved, no hematuria	Chemotherapy: Nab-paclitaxel 0.3g d1 q3w ×2 cycles Immunotherapy: Tislelizumab 200mg d1 q3w ×2 cycles	No new adverse reactions	2023–01 CT: Pulmonary metastases sustained PR (partial complete regression) Renal primary lesion shrank to 8.5×7.7 cm → PR
2023-03 — 2023-08	Asymptomatic	Immunotherapy maintenance: Tislelizumab 200mg q3w	No new adverse reactions	Sustained PR (no progression shown)
2023-09	Sudden gross hematuria, oliguria	Self-administration of traditional Chinese medicine	Acute kidney injury: Creatinine 567.1 μmol/L, hyperkalemia	CT confirmed disease progression: Left renal hilar lymph node enlargement >20% → PD
2023-11	Anuria, multiple organ failure	Dialysis support therapy	End-stage renal disease	Death, overall survival 14 months

## 3 Discussion

In this study, we present a rare case of squamous cell carcinoma characterized by a space-occupying lesion in the left kidney, accompanied by bilateral lung metastases. The identification of the primary site has been a subject of considerable debate. Imaging findings suggest a primary tumor in the left kidney with venous tumor thrombus; however, histological confirmation could not be obtained due to the risks associated with biopsy. Conversely, the pathologically confirmed lung squamous cell carcinoma raises the possibility of either a primary lung tumor metastasizing to the kidney or a primary renal carcinoma metastasizing to the lungs, without ruling out the potential for dual primary sites. Following a comprehensive MDT evaluation, and based on the principles of monism, imaging characteristics, tumor biological behavior, and analysis of metastatic patterns, the final diagnosis was established as primary renal squamous cell carcinoma with bilateral lung metastases. The primary diagnostic criteria are as follows:

Primary renal squamous cell carcinoma (PRSCC) accounts for less than 1% of urological malignancies and is characterized as a rare and highly aggressive tumor with a poor prognosis. PRSCC predominantly originates from the renal pelvis (10), while its occurrence in the renal parenchyma is relatively uncommon (11). In this case, the renal lesion was situated within the renal parenchyma, which is inconsistent with the typical primary site distribution of renal squamous cell carcinoma. In contrast to the rarity of renal squamous cell carcinoma, lung squamous cell carcinoma (LSCC) is the second most common histological subtype of non-small cell lung cancer, following adenocarcinoma (12, 13). LSCC generally originates in the central regions of the lungs or major airways (14). However, in the present case, pulmonary lesions were predominantly located in peripheral regions, which contradicts the typical primary site preference associated with LSCC. If anatomical localization discrepancies cannot definitively determine the primary origin of squamous cell carcinoma in this instance, can reliance solely on the incidence disparity between LSCC and RSCC suffice to establish the diagnosis? Although LSCC has a significantly higher incidence than RSCC, their metastatic patterns exhibit distinct biological differences. The most common metastatic sites for lung cancer include the brain and bones, whereas solitary renal metastasis from primary pulmonary squamous cell carcinoma is exceedingly rare (15). Moreover, renal metastases typically present as small, multifocal, bilateral, wedge-shaped, endophytic lesions confined within the renal capsule (16). In stark contrast, the renal lesion in this case demonstrated characteristics that were fundamentally different: it was a large (9.4×7.9 cm), solitary, unilateral mass confined within the renal parenchyma, markedly deviating from the typical radiological features associated with metastatic renal involvement. Furthermore, in the cohort of lung cancer patients with renal metastases studied by Adamy et al., only 15% exhibited gross hematuria, with the majority of renal metastatic lesions remaining asymptomatic (17). Notably, the present case displayed prominent symptoms of gross hematuria and flank pain, while completely lacking pulmonary symptoms, thereby further diminishing the likelihood of a primary pulmonary origin. In contrast, the metastatic profile of renal carcinoma closely aligns with the current case. The lung is the most common site for metastasis in renal carcinoma, accounting for 45% of metastatic renal cell carcinoma cases, with pulmonary metastases typically remaining asymptomatic (5). Sas S et al. reported that among 10 incidentally diagnosed cases of RSCC, two exhibited metastatic pulmonary nodules (18). This discrepancy in metastatic patterns, coupled with contrasting clinical presentations, provides critical evidence supporting the diagnostic orientation towards a primary renal origin with pulmonary metastases.

The clinical diagnosis and management of PRSCC continue to present significant challenges, as no standardized guidelines or treatment protocols have been established to date. Radical surgery remains the primary therapeutic option for early- and intermediate-stage PRSCC (19). Given that the majority of renal squamous cell carcinomas are diagnosed at advanced stages (20), characterized pathologically by moderately to poorly differentiated histology, the prognosis remains poor, with 5-year survival rates below 10% (21). This underscores the urgent need to explore novel therapeutic strategies. In recent years, immune checkpoint inhibitors (ICIs) have made groundbreaking advancements in the treatment of renal cell carcinoma (RCC). The KEYNOTE-564 trial has shown that adjuvant pembrolizumab significantly extends disease-free survival in patients with high-risk clear cell RCC (ccRCC) (22) Regrettably, there remains an absence of standardized treatment protocols for RSCC. At present, there are no documented cases of immunotherapy application for PRSCC, and the available evidence is confined to small-scale investigations concentrating on non-clear cell renal cell carcinoma (nccRCC) subtypes (23). Among the most extensive prospective trials conducted thus far is Cohort B of the KEYNOTE-427 trial. This multicenter, non-randomized phase II study involved 165 patients with advanced nccRCC who received pembrolizumab monotherapy as a first-line treatment, resulting in an objective response rate (ORR) of 26.7% in the overall population and a median progression-free survival (mPFS) of 4.2 months (24). Similarly, the CheckMate 374 trial demonstrated that nivolumab monotherapy achieved favorable anti-tumor efficacy in patients with advanced nccRCC, with an ORR of 23% and an mPFS of 4.0 months (25). Furthermore, a meta-analysis conducted by the Petrelli group, which encompassed 23 studies on first-line immunotherapy for various nccRCC subtypes, corroborated the efficacy of ICIs, reporting a pooled ORR of 26.6% and a median PFS of 6.59 months (26). Nonetheless, Nevertheless, none of these studies specifically incorporated the RSCC subtype in their analyses. Concurrently, other research indicates that patients with nccRCC may experience less favorable survival outcomes from immune checkpoint inhibitor (ICI) monotherapy compared to those with ccRCC (25, 27–30). A study conducted by Bimbatti et al. further corroborated the significant variations in treatment efficacy among nccRCC subtypes. They categorized nccRCC into papillary renal cell carcinoma (pRCC), renal cell carcinoma not otherwise specified (NOS-RCC), and other histological types, discovering that the mPFS following ICI treatment was 9.7 months for pRCC patients, whereas it was merely 3.0 months for other nccRCC subtypes (31). These disparate findings suggest that the effectiveness of ICIs in treating nccRCC is highly contingent upon the tumor’s histological subtype and inherent heterogeneity. Nevertheless, current evidence suggests that patients with nccRCC may experience a relatively high ORR with first-line ICI therapy (28, 29). Furthermore, research has demonstrated that patients exhibiting positive programmed death-ligand 1 (PD-L1) expression derive significantly greater benefit from ICI treatment compared to those with negative PD-L1 expression (32–34). This correlation has been substantiated by large-scale studies; for instance, in a cohort of 8,887 patients analyzed by Christopher et al., the degree of therapeutic benefit was markedly higher in individuals with elevated PD-L1 expression (P = 0.006) (35). Consequently, PD-L1-positive patients may achieve the greatest survival advantage from adjuvant immunotherapy (36, 37), thereby providing a theoretical foundation for the use of ICIs in PRCC. However, clinical decision-making must holistically consider factors such as tumor histological subtype, patient physical status, comorbidities, and clinical manifestations (27).

This case involves a 65-year-old male patient diagnosed with renal squamous cell carcinoma (cT3N2M1, stage IV), which is complicated by lung metastasis and a tumor thrombus in the left renal vein. The patient’s Eastern Cooperative Oncology Group (ECOG) performance status is 1, and he has a medical history that includes hypertension, diabetes, and coronary atherosclerotic heart disease, rendering him inoperable. Currently, there is no established systemic treatment protocol for metastatic renal squamous cell carcinoma. In light of the rarity of such cases, Sas S et al. have proposed a treatment regimen consisting of cisplatin-based chemotherapy combined with palliative radiotherapy for similar advanced cases (18), which aligns with our initial treatment strategy. However, the survival benefit associated with this regimen remains uncertain, as none of the participants in their study elected to undergo chemotherapy. In light of the absence of standardized systemic treatment protocols for nccRCC, encompassing RSCC, combined with the patient’s surgical contraindications, an individualized treatment strategy was developed after thorough deliberations by a MDT. Given the patient’s compromised general health and multiple comorbidities, which could potentially lead to intolerance of cisplatin chemotherapy’s side effects, single-agent albumin-bound paclitaxel was selected. Concurrently, palliative radiotherapy targeting the renal lesion was incorporated to alleviate hematuria and mitigate the risk of tumor thrombus detachment. Genetic testing revealed a PD-L1 tumor proportion score (TPS) of 55%, indicating a potential benefit from immunotherapy. Although pembrolizumab was initially recommended, tislelizumab was ultimately selected due to the patient’s financial limitations. Following two cycles of combined chemo-immunotherapy and radiotherapy, subsequent chest CT scans demonstrated significant regression of pulmonary lesions (**Figure 1D**). Continued triweekly immunotherapy over two months resulted in further reduction, including complete resolution of certain lesions (left panel, **Figure 1F**). The overall response was classified as a PR according to the RECIST v1.1 criteria.

Following radiotherapy, the primary renal lesion exhibited resolution of hematuria and flank pain; however, the extent of tumor reduction did not satisfy the criteria for PR. This outcome contrasts sharply with the significant regression observed in pulmonary metastatic lesions, a discrepancy that may be attributed to the inherent radioresistance of RCC to conventional fractionated radiotherapy (38). Funayama et al. reported that even with the application of stereotactic body radiation therapy (SBRT), which is known for its potent cytotoxic effects, renal tumor responses were notably slow. Among 13 RCC patients studied, the majority achieved PR, but only after a gradual reduction in tumor volume over extended periods, with a median time to confirmed PR of 22.6 months for partial or complete responders (39). In the present case, the primary renal lesion demonstrated a 14.3% reduction in volume (from 9.4×7.9 cm to 8.5×7.7 cm) within 4 months, aligning with the criteria for Grade II radiosensitivity classification.

The notable regression of pulmonary metastatic lesions may result from the synergistic interactions of multiple mechanisms. The primary focus is the radiation-induced abscopal effect, a biological phenomenon first described by Mole in 1953, characterized by the spontaneous regression of unirradiated distant metastases following localized radiotherapy (40). The incidence of the abscopal effect varies considerably across different tumor types. A meta-analysis conducted by Steven et al. revealed that approximately two-thirds of reported abscopal effects were concentrated in specific malignancies, such as renal carcinoma, non-small cell lung cancer, and melanoma (41). Of greater clinical significance is the systematic review by Abuodeh et al., which examined globally reported cases from 1969 to 2014. Among the 46 cases of the abscopal effect, three patients with RCC exhibited pulmonary metastatic regression following irradiation of the primary renal tumor (42), a pattern that closely resembles the clinical course observed in the present case.

At the mechanistic level, radiotherapy induces immunogenic cell death (ICD) in tumor cells, leading to the release of tumor-associated antigens (TAAs) and the activation of inflammatory signaling pathways, which collectively initiate systemic anti-tumor immune responses. Nevertheless, the incidence of abscopal effects resulting from radiotherapy alone remains below 5%. An increasing body of research suggests that the combination of radiotherapy with immunotherapy markedly enhances abscopal responses (40). Specifically, PD-1/PD-L1 blockade reverses T-cell exhaustion and amplifies radiation-induced systemic immunity. This synergistic approach facilitates the migration of tumor-reactive T cells to non-irradiated sites, thereby inducing regression of secondary tumors beyond the radiation field and achieving effective control of distant metastases (43, 44). In the current case, the concurrent administration of the PD-1 inhibitor tislelizumab during radiotherapy likely mediated the observed abscopal effect through these mechanisms. While radiation and immunotherapy may have predominantly contributed to the regression of pulmonary metastases, the synergistic impact of nab-paclitaxel should not be underestimated. As a classic chemotherapeutic agent, the paclitaxel family is extensively employed in cancer treatment due to its strong microtubule-stabilizing properties (45). In comparison to conventional paclitaxel formulations, nab-paclitaxel utilizes the enhanced permeability and retention (EPR) effect to achieve greater tumor-targeted accumulation. This not only augments antitumor efficacy but also significantly enhances drug safety and tolerability (46). Therefore, the pronounced response of pulmonary lesions observed in this case is likely attributable to the integrated mechanisms of radiotherapy, immunotherapy, and chemotherapy.

The patient experienced acute kidney injury (AKI) 12 months following the administration of combined chemoradiotherapy and ICI therapy, subsequently succumbing to the condition 2 months thereafter. A comprehensive analysis of the etiology of AKI in this case necessitates consideration of several multidimensional factors. Firstly, a large-scale meta-analysis conducted by Amisha et al., encompassing 48 clinical studies with a total of 11,482 participants, reported an overall incidence of AKI following PD-1 inhibitor therapy at merely 2.2% (47). The median onset of ICI-related AKI (ICPi-AKI) was identified at 16 weeks post-treatment (IQR 8–32 weeks). In this particular case, AKI manifested 48 weeks (12 months) after the initiation of treatment, representing a delay of 32 weeks beyond the typical onset period, thereby attenuating the causal link to ICPi-AKI. Secondly, from a pathological perspective, acute interstitial nephritis (AIN) is the predominant histological manifestation of ICI-related toxicity (48). However, this patient lacked renal biopsy histopathological evidence or key indicators such as eosinophilic infiltration, making it difficult to directly attribute AKI to immune toxicity. Merza’s study identified a history of hypertension as an independent risk factor for AKI (OR 4.3; 95% CI 1.8–6.1; p<0.001). Notably, AKI was found to be independent of the type of ICI drug or cancer type (49). This particular patient had a 10-year history of hypertension, coronary artery disease, and diabetes, suggesting that the underlying renal injury due to chronic hemodynamic alterations should not be overlooked. Furthermore, the patient consistently self-administered complex traditional Chinese medicine formulations during treatment, which may have contained nephrotoxic components that exacerbated renal dysfunction through drug-drug interactions. Additionally, the concurrent progression of the primary renal lesion at the onset of AKI raises the possibility of tumor infiltration or factors related to paraneoplastic syndrome. In conclusion, the etiology of AKI in this patient is likely attributable to multifactorial interactions among preexisting comorbidities, pharmacological toxicity, and tumor progression.

In light of the fact that the majority of RSCC cases documented in the current literature have been treated surgically, and there is a paucity of directly comparable cases involving combined chemoradiotherapy with immunotherapy or immunotherapy alone (50, 51), this study employed an indirect comparison approach. Sas et al. documented 14 RSCC patients who underwent surgical treatment without immunotherapy, reporting a median OS of merely 5 months (18). In contrast, the patient with advanced-stage RSCC in this study received combined chemoradiotherapy with immunotherapy, resulting in an OS of 14 months, which is nearly threefold longer than that associated with the conventional treatment approach. Furthermore, when compared to other rare subtypes of nccRCC, Li et al. described a case involving a 60-year-old male patient with collecting duct carcinoma (CDC, with an incidence of approximately 1%), who exhibited PD within 1 month and achieved an OS of less than 2 months following treatment with chemotherapy, camrelizumab immunotherapy, and sorafenib targeted therapy (52). In conclusion, this case illustrates that the integration of the radiotherapy-induced abscopal effect with the synergistic impact of chemotherapy-immunomodulation substantially extended the survival of patients with advanced RSCC, suggesting a promising new treatment paradigm for inoperable cases. Nevertheless, the findings of this study are derived from the treatment experience of a single patient; thus, the generalizability and long-term efficacy of these results require further validation through cohort studies with larger sample sizes.

## 4 Conclusion

In conclusion, renal squamous cell carcinoma (RSCC) is an uncommon malignancy. For patients who are not candidates for surgical intervention, the combination of chemoradiotherapy and PD-1 inhibitors has shown promising efficacy, potentially extending survival and improving prognosis to a certain degree. This approach may serve as a valuable reference for the comprehensive management of advanced RSCC, although vigilant monitoring for nephrotoxicity is essential.

## Data Availability

The datasets presented in this study can be found in online repositories. The names of the repository/repositories and accession number(s) can be found in the article/supplementary material.
