# Correction: Case Report: The integration of chemoradiotherapy and immunotherapy in a patient with advanced-stage renal squamous cell carcinoma and pulmonary metastases

**DOI:** 10.3389/fimmu.2025.1751999

**Published:** 2025-11-28

**Authors:** Yanlin Niu, Shengchao Wang, Xinzhou Deng, Ming Luo, Jingjing Chai, Zhiguo Luo

**Affiliations:** 1 Department of Oncology, Taihe Hospital, Hubei University of Medicine, Shiyan, Hubei, China; 2 Key Laboratory of Cancer Therapy Resistance and Clinical Translational Study, Shiyan, Hubei, China

**Keywords:** renal squamous cell carcinoma, pulmonary metastases, immunotherapy, radiotherapy, chemotherapy

“Affiliation 1: Department of Oncology, Taihe Hospital, Hubei University of Medicine, Shiyan, Hubei, China” was omitted from the paper. This affiliation has now been added for author(s) Shengchao Wang.

The original version of this article has been updated.

